# Interim results of an ongoing Phase I, dose escalation study of MGA271 (Fc-optimized humanized anti-B7-H3 monoclonal antibody) in patients with refractory B7-H3-expressing neoplasms or neoplasms whose vasculature expresses B7-H3

**DOI:** 10.1186/2051-1426-3-S2-O8

**Published:** 2015-11-04

**Authors:** John Powderly, Gregory Cote, Keith Flaherty, Russell Z Szmulewitz, Antoni Ribas, Jeffrey Weber, Deryk Loo, Jan Baughman, Francine Chen, Paul Moore, Ezio Bonvini, James Vasselli, Jon Wigginton, Roger Cohen, Howard Burris, Bartosz Chmielowski

**Affiliations:** 1 Carolina BioOncology Institute, Huntersville, NC, USA; 2 Department of Oncology, Massachusetts General Hospital and Harvard Medical School, Boston, MA, USA; 3 University of Chicago, Chicago, IL, USA; 4 University of California at Los Angeles Medical Center, Los Angeles, CA, USA; 5 H. Lee Moffitt Cancer Center, Tampa, FL, USA; 6 MacroGenics, San Francisco, CA, USA; 7 MacroGenics, Rockville, MD, USA; 8 University of Pennsylvania, Philadelphia, PA, USA; 9 Sarah Cannon Research Institute, Nashville, TN, USA; 10 Division of Hematology - Medical Oncology, UCLA Jonsson Comprehensive Cancer Center, Los Angeles, CA, USA

## Background

MGA271 is a humanized IgG1 monoclonal antibody-targeting B7-H3 (CD276), a member of the B7 family. MGA271 has been Fc-engineered to enhance binding to activating FcγR (CD16A), decrease binding to inhibitory FcγR (CD32B), and potentiate ADCC. B7-H3 has limited expression in normal tissues but highly expressed in a broad range of tumors, making it an attractive target for cancer immunotherapy. We initiated a Phase I investigation of the safety, tolerability, pharmacokinetics, pharmacodynamics and antitumor activity of MGA271.

## Methods

This Phase I study included dose escalation (complete) and an ongoing two-component cohort expansion (NCT01391143). Dose escalation utilized single-patient, intra-patient escalation, followed by a conventional 3+3 design, using MGA271 doses of 0.01-15mg/kg. MGA271 was administered weekly on a 4-week on, 4-week off schedule during cycle 1, and 3 out of every 4 weeks in subsequent cycles. Initial expansion cohorts enrolled patients (N=15/cohort) with melanoma, prostate carcinoma and other B7-H3+ tumors. Major modifications were subsequently made to enhance the functionality of the study prior to enrolling additional expansion cohorts. MGA271 administration was changed to uninterrupted weekly dosing, corticosteroid infusion reaction prophylaxis was reduced and immune-related response criteria and management principles were implemented. The additional expansion cohorts (N=16/cohort) were initiated in patients with melanoma (post checkpoint inhibitor), squamous cell head and neck carcinoma, renal cell carcinoma, triple-negative breast carcinoma, and high-B7-H3 expressing tumors including bladder and lung carcinoma. Pharmacodynamic testing included serum cytokines and modulation of the lymphocyte phenotype and T cell repertoire in peripheral blood.

## Results

MGA271 was well tolerated, with no dose-limiting toxicity and no maximum tolerated dose defined up to 15 mg/kg. Patients were frequently heavily pre-treated, with a median of 3 (0-7) prior therapies. Any grade treatment-related AEs occurred in 71% of patients, including fatigue (30%), infusion-related reaction (26%), nausea (19%), chills (17%) and vomiting (13%). Grade 3/4 drug related AEs were noted in 6% of patients. No drug-related AEs led to study drug discontinuation. MGA271 showed linear PK. As of 30 July 2015, 20 of 46 patients treated under the new study design continue on study drug. Patients experienced disease stabilization (>12 weeks) and tumor shrinkage (2-69%) across several tumor types. The study continues to enroll patients and generate more mature data.

## Conclusions

MGA271 has a favorable safety profile and initial evidence of anti-tumor activity across several tumor types. These data support continued evaluation of MGA271 monotherapy and in combination with other immune modulators, including checkpoint inhibitors.

## Trial Registration

ClinicalTrials.gov Identifier NCT01391143

